# Development of an optically stimulated luminescence radiometer based on sodium chloride and gold nanoparticles for improving radiation detection in radiography

**DOI:** 10.1002/acm2.70539

**Published:** 2026-05-01

**Authors:** Mus'ab S Alkasasbeh, Khalid Hassan Ibnaouf, Naser M Ahmed, Azhar Abdul Rahman, Thair Hussein Khazaalah, Dheyaa Nabeel Abbas, Hajo Idriss, Monther Alsboul

**Affiliations:** ^1^ Department of Allied Medical Sciences Faculty of Applied Medical Sciences Al al‐Bayt University Mafraq Jordan; ^2^ Department of Physics College of Science Imam Mohammad Ibn Saud Islamic University (IMSIU) Riyadh Saudi Arabia; ^3^ Laser and Optoelectronics Engineering Department Dijlah University Baghdad Iraq; ^4^ School of Physics Universiti Sains Malaysia Penang Malaysia; ^5^ Faculty of Education Universiti Teknologi MARA, Puncak Alam Branch Bandar Puncak Alam Selangor Malaysia; ^6^ Physics Department College of Science, Al‐Hussein Bin Talal University Ma'an Jordan

**Keywords:** detectors, dosimetry, measurement of radiation, NaCl, OSL

## Abstract

**Purpose:**

To evaluate sodium chloride doped with gold nanoparticles (NaCl + GNPs) as a low‐cost, reusable optically stimulated luminescence (OSL) material for diagnostic radiology, and to benchmark its luminescence performance against the commercial standard, aluminum oxide doped with carbon (Al_2_O_3_:C).

**Methods:**

NaCl + GNPs detectors were fabricated through solution‐based doping, solvent evaporation, pellet pressing, and two‐stage thermal treatment. Structural characterization was verified via UV–Vis spectroscopy. OSL performance was assessed under diagnostic X‐ray energies ranging from 40 to 150 kVp, using Al_2_O_3_:C as the reference. Evaluated parameters included OSL signal linearity, reproducibility, bleaching efficiency, fading behavior, minimum detectable signal (MDS), energy dependence, and mass scaling. Measurements were performed using equal and variable detector masses (20–60 g) across a dose range of approximately 6–60 mGy.

**Results:**

NaCl + GNPs exhibited strong linearity (*R* ^2^ > 0.99) across the tested exposure range and produced reproducible OSL signals over repeated irradiations. The material demonstrated an energy‐dependent increase in luminescence comparable to Al_2_O_3_:C, with the 40 g NaCl + GNPs sample achieving 96–97% of the Al_2_O_3_:C OSL signal at 150 kVp. Increasing detector mass enhanced luminescence output proportionally, with 60 g samples exceeding the reference material at energies above 100 kVp. Bleaching efficiency was notably high, with consistently minimal residual signal, confirming excellent reusability. The MDS was approximately equivalent to 0.4–0.5 mGy in relative terms. However, NaCl + GNPs showed faster fading, with ∼50% signal loss over 9 h, indicating the importance of timely readout or controlled storage.

**Conclusions:**

Gold nanoparticle incorporation enhances the luminescent efficiency and usability of NaCl, producing a material that is low‐cost, scalable, and highly effective in bleaching. Although slightly less sensitive than Al_2_O_3_:C, the NaCl + GNPs detectors demonstrate strong performance, favorable reproducibility, and minimal residual signal, making them a practical option for clinical, educational, and resource‐limited dosimetry applications.

## 1 INTRODUCTION

Accurate radiation dose monitoring is essential in diagnostic radiology to ensure both patient safety and adherence to regulatory standards. Optically stimulated luminescence (OSL) has emerged as a preferred dosimetry technique due to its high sensitivity, reusability, and wide dynamic range.^1^, ^2^ Among the established OSL materials, aluminum oxide doped with carbon (Al_2_O_3_:C) has gained widespread acceptance for its excellent dosimetric performance.^3^ Nevertheless, its commercial production remains costly and technically demanding, limiting its accessibility in resource‐limited settings.^4^ In the search for alternative materials, sodium chloride (NaCl) has garnered attention due to its natural abundance, low cost, and inherent luminescent properties under ionizing radiation.^5^ NaCl, when optically stimulated, exhibits a measurable luminescence signal that makes it a potential candidate for OSL applications.^6^ Furthermore, its chemical stability and non‐toxicity render it an attractive choice for radiation dosimetry in medical environments.^7^ However, pure NaCl has historically demonstrated limited sensitivity and requires enhancement to meet the performance standards of commercial detectors.^8^ Recent advances in nanotechnology have opened new avenues for improving the luminescence properties of dosimetric materials. Among these, the incorporation of gold nanoparticles (GNPs) has shown promise in enhancing radiation interaction and luminescence yield due to localized surface plasmon resonance (LSPR) effects.^9^, ^10^ GNPs exhibit unique electromagnetic field enhancement properties, which can lead to increased charge carrier generation and improved signal intensity in OSL materials.^11^

One of the key motivations for developing NaCl‐based OSL detectors is their significantly lower fabrication cost compared to commercial Al_2_O_3_:C nanoDots. A single Al_2_O_3_:C detector typically costs USD 8–12 per chip depending on the supplier and shipping restrictions. In contrast, the material cost of a NaCl + GNPs pellet is less than USD 0.03–0.05 (including NaCl, GNP solution, and processing consumables). This represents a reduction in cost by a factor of approximately 200–300×, making NaCl + GNPs an attractive solution for large‐scale dose monitoring, educational settings, and low‐resource environments.

While GNPs have been investigated in combination with various host matrices, such as glass, phosphors, and polymer composites,^12^, ^13^ limited research has explored their integration with NaCl for dosimetric purposes.^14^ To date, no comprehensive studies have evaluated the performance of a NaCl‐based OSL detector doped with GNPs as a viable alternative to Al_2_O_3_:C. This represents a clear gap in the literature. Existing investigations have largely focused on NaCl as a standalone material^5^, ^6^, ^7^ or on the use of nanoparticles in general, without examining the synergistic effect of NaCl combined with GNPs under clinical X‐ray exposures.^14^ Moreover, previous studies have not systematically compared the response of such novel composites to that of the gold standard, Al_2_O_3_:C, under a range of diagnostic kilovoltage peaks (kVp), which are critical for real‐world radiographic applications.^15^

GNPs exhibit a phenomenon known as LSPR, where the conduction electrons on the nanoparticle surface oscillate collectively when excited by incident electromagnetic radiation. This oscillation produces intense local electromagnetic fields around the nanoparticle, significantly increasing the rate of electron–hole pair generation within the surrounding host material. In the context of OSL dosimetry, these enhanced local fields improve charge trapping efficiency and increase the probability of radiative recombination during optical stimulation. Because Al_2_O_3_:C does not possess plasmonic behavior, the addition of GNPs introduces a unique signal‐enhancement mechanism that is not available in conventional commercial OSL materials.

In this study, we present the design and development of an optically stimulated scintillation radiometer based on NaCl doped with GNPs. We investigate its fabrication, structural properties, and radiation response under various X‐ray energies, and compare its performance to that of Al_2_O_3_:C. This research aims to demonstrate that NaCl + GNPs offers a safe, low‐cost, and effective alternative for radiation dose monitoring, with potential applications in both patient and occupational dosimetry.

## 2 MATERIALS AND METHODS

### 2.1 Materials selection and preparation

Analytical‐grade NaCl was selected as the base material due to its high purity, nontoxic nature, low cost, and inherent luminescent properties. GNPs were used as a dopant to enhance the luminescence yield via LSPR. A colloidal gold nanoparticle solution with an average particle diameter of 20 nm was utilized for optimal interaction with the NaCl matrix and improved energy absorption. To ensure consistent stoichiometry and uniform distribution of nanoparticles, precise quantities of NaCl were measured using a high‐resolution analytical balance (± 0.1 mg accuracy). The weighed NaCl was dissolved in deionized water and mixed thoroughly with a calculated volume of GNP colloidal suspension. This blending step was carried out under magnetic stirring for 2 h at room temperature to ensure complete homogenization of the nanoparticle dispersion within the salt matrix.

**FIGURE 1:**
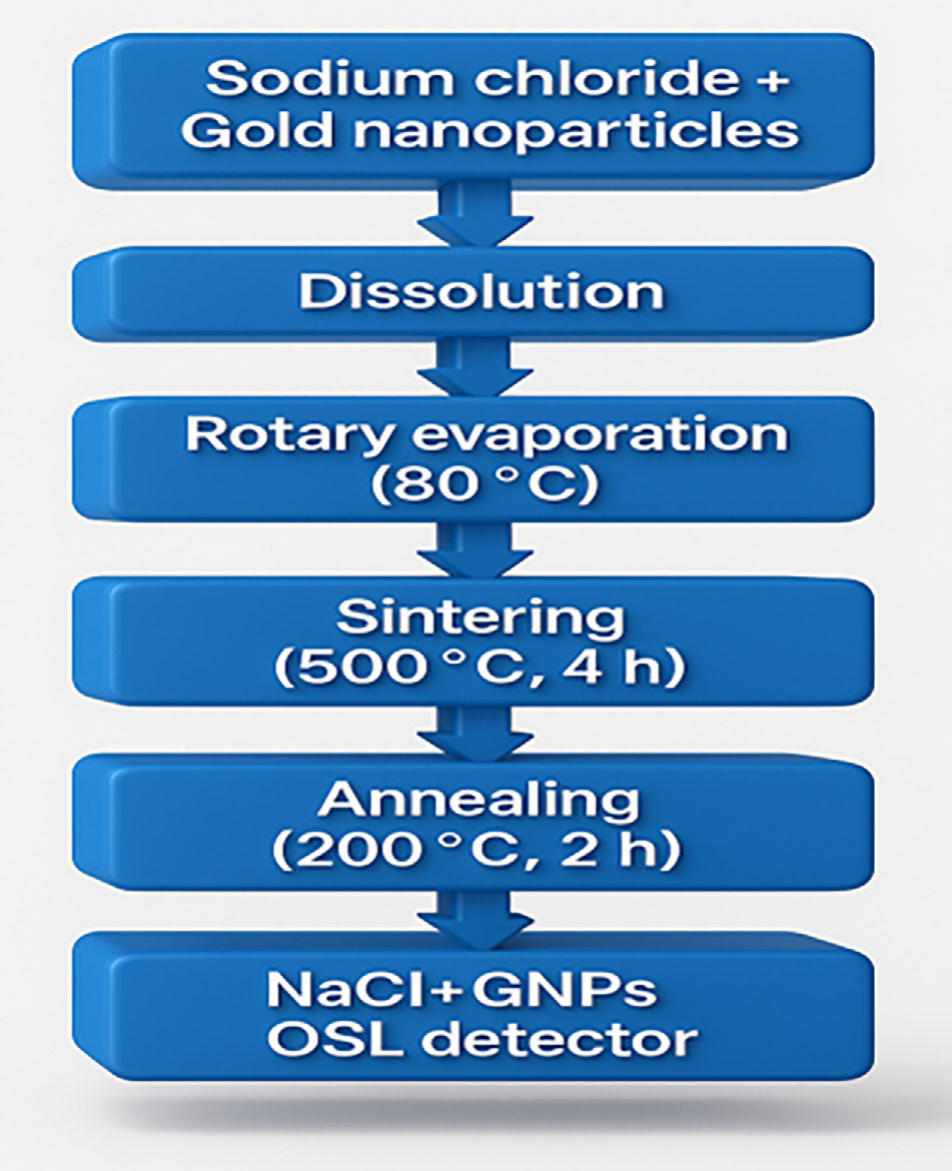
Schematic diagram of the synthesis process for NaCl + GNPs‐based OSL detector.

**FIGURE 2:**
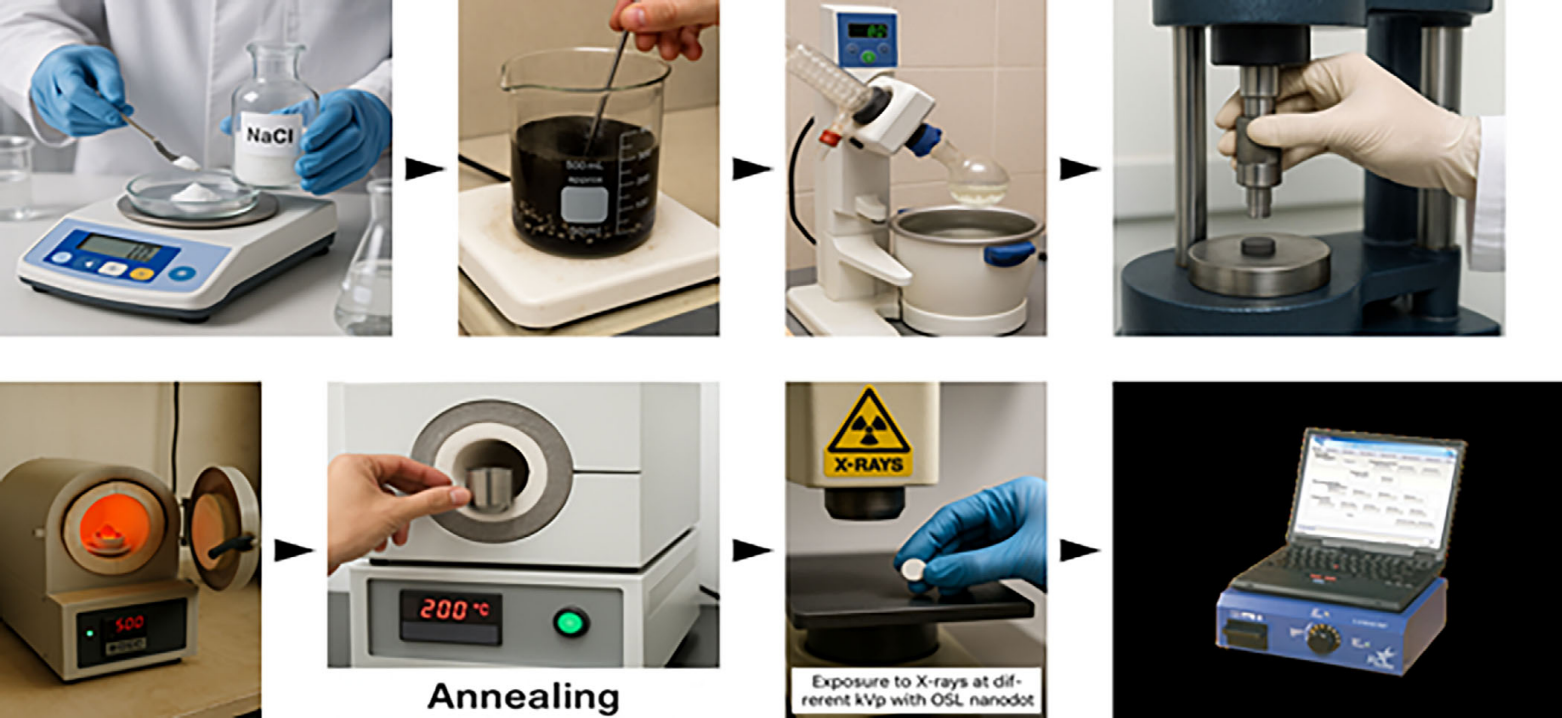
Photographic representation of the experimental workflow for NaCl + GNPs OSL detector preparation and testing.

In the drying and solid‐state conversion, the resulting NaCl‐GNPs solution was subjected to rotary evaporation using a Büchi R‐300 evaporator set at 80°C under reduced pressure. This step ensured the removal of residual solvent and produced a dry, homogeneously doped NaCl powder free from agglomerations and impurities. The Pellet Formation and Sintering. The dried NaCl + GNPs powder was pressed into uniform circular pellets using a hydraulic press (Specac Atlas™) under a load of 5 tons for 2 min. Pellets were formed with a diameter of 13 mm and a thickness of approximately 1.5 mm to standardize the OSL measurement geometry.To enhance crystallinity and densify the structure, the pellets were thermally treated in a high‐temperature tube furnace. A two‐stage thermal process was employed:

‐ Sintering: Performed at 500°C for 4 h under a continuous flow of ultra‐high‐purity nitrogen gas (99.999%) to prevent oxidation and maintain chemical stability(Figure 1).

‐ Annealing: Conducted at 200°C for 2 h to relieve internal lattice stresses and optimize the trap structure responsible for OSL signal storage and release. The pellet dimensions (13 mm diameter and ∼1.5 mm thickness) were selected based on mechanical stability during pressing, compatibility with the microStar OSL reader tray, and the requirement to provide a sufficiently large surface area for uniform optical stimulation during readout. These dimensions differ from the commercial Al_2_O_3_:C nanoDot (approximately 4 mm in diameter and 0.3 mm in thickness), primarily because NaCl‐based detectors require a larger pellet mass to achieve adequate luminescence output due to their lower intrinsic sensitivity. A comparative photograph of both detectors is shown in Figure 2 to visually illustrate the dimensional differences and justify the selected geometry.

The selected fabrication route—solution‐based mixing followed by solvent evaporation, pellet pressing, and controlled two‐stage thermal treatment—has been widely adopted in the preparation of halide‐based OSL materials and nanoparticle‐doped phosphors. Previous studies have demonstrated that this processing sequence improves structural homogeneity, trap stability, and overall luminescent efficiency in NaCl‐based detectors and composite OSL systems.^5^, ^12^, ^20^ These references support the suitability of the adopted method and justify its application in the present work.

This thermal treatment sequence was critical in promoting grain growth and increasing the concentration of luminescence‐relevant defects within the NaCl lattice. The Reference Material: Al_2_O_3_:C and for benchmarking, commercially prepared Al_2_O_3_:C (Landauer, Inc.) chips were used as the reference OSL material. These detectors are known for their high sensitivity and stability and were subjected to the same irradiation and readout procedures to ensure direct comparability. OSL measurements were conducted using a calibrated microStar OSL reader (Landauer, USA), ensuring precise and consistent signal acquisition across all evaluated samples.

The fabrication steps include dissolution of NaCl and GNPs, rotary evaporation at 80°C for solvent removal, pellet pressing, sintering at 500°C for 4 h under nitrogen atmosphere, followed by annealing at 200°C for 2 h. These sequential thermal and mechanical treatments enable the formation of a stable, luminescent NaCl + GNPs pellet optimized for OSL dosimetry.

Figure 2 illustrates the sequential experimental steps: precise weighing of NaCl, nanoparticle mixing and solution homogenization, rotary evaporation at 80°C for drying, pellet pressing using a hydraulic press, sintering at 500°C, annealing at 200°C, X‐ray irradiation at various kVp levels, and final OSL signal readout using a calibrated reader. This workflow demonstrates the reproducibility and laboratory feasibility of synthesizing the NaCl + GNPs composite for radiation dosimetry applications.

### 2.2 UV‐vis analysis

**FIGURE 3:**
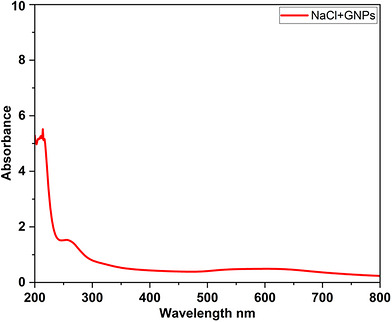
UV–vis absorption spectrum of the NaCl + GNPs composite, indicating surface plasmon resonance behavior.

Figure 3 shows the UV‐Vis absorption spectrum of the NaCl + GNPs sample, as presented in the figure, displays a prominent absorption peak in the deep ultraviolet region, specifically around 200–230 nm. This intense absorbance is attributed to the strong electronic transitions within the NaCl matrix, likely due to n → σ or π → π transitions associated with its crystalline structure and the presence of surface defects introduced during doping. Following this sharp peak, the absorbance decreases rapidly, indicating that the material becomes increasingly transparent in the visible region (400–700 nm). A subtle shoulder or broad hump is observed around 520–540 nm, which corresponds to the LSPR of the embedded GNPs. This LSPR band is a key optical signature of gold nanostructures and confirms their successful incorporation into the NaCl host. The overall spectral behavior highlights the material's high sensitivity to UV light and its minimal absorbance in the visible range an advantageous combination for OSL applications. Specifically, the strong UV absorption facilitates efficient excitation, while the transparency in the visible spectrum ensures low background interference during signal readout. These optical properties support the suitability of NaCl + GNPs for use in radiation dosimetry, especially in low‐dose medical imaging environments such as computed tomography (CT).

While Figure 3 reports the optical absorption characteristics of the NaCl + GNPs samples, the emission band is also of critical importance for OSL readout. Pure NaCl is known to exhibit blue‐region luminescence with emission peaks typically between 350 and 420 nm, depending on impurities and defect centers. The incorporation of GNPs can enhance the emission intensity through plasmonic amplification, but does not significantly shift the primary emission wavelength. In comparison, Al_2_O_3_:C exhibits a well‐defined emission peak at ∼410 nm, which closely matches the photomultiplier tube (PMT) sensitivity and optical filtering of the microStar reader. Therefore, any slight spectral mismatch between NaCl + GNPs emission and the 410 nm detection window may contribute to reduced signal efficiency. This factor is considered in the interpretation of sensitivity differences reported in this work.

### 2.3 OSL readout procedure and calibration

All measurements were performed using a Landauer microStar reader operating in continuous‐wave OSL mode. Prior to experimental measurements, the reader was calibrated using the manufacturer's standard calibration protocol with a commercial Al_2_O_3_:C nanoDot irradiated at 80 kVp on a GE diagnostic X‐ray system. The calibration ensured repeatable luminescence output and established the reader's PMT gain and stimulation power settings. The calibration factor supplied by the system was applied uniformly to all subsequent measurements.

Because the NaCl + GNP pellets (13 mm × ∼1.5 mm) differ in geometry from the commercial nanoDots (4 mm × 0.3 mm), several steps were taken to ensure consistent light collection. First, each pellet was centered in the microStar sample tray to maximize uniform optical stimulation. Second, the larger surface area of the NaCl pellets was accounted for by normalizing all signals to the exposed mass, ensuring a fair comparison with the reference Al_2_O_3_:C material. The stimulation wavelength (blue LEDs, ∼470 nm) and PMT sensitivity range (∼350–500 nm) were confirmed to be compatible with the emission characteristics of NaCl + GNPs. This expanded description ensures accurate interpretation of the readout conditions and the influence of detector geometry.

### 2.4 Diagnostic X‐ray system and dosimetry calibration

All irradiations were carried out using a GE Definium 5500 diagnostic radiography system (GE Healthcare, USA). The X‐ray tube features inherent filtration of 2.5 mm Al with additional filtration provided by the system's automatic beam‐hardening configuration. The tube voltage was varied between 40 and 150 kVp in 10–20 kVp increments. Exposures were delivered using tube currents between 50 and 200 mA, with a fixed source‐to‐detector distance (SID) of 110 cm and a field size of 20 × 20 cm to ensure uniform beam coverage over all OSL samples.

To ensure consistency and accuracy, beam output was calibrated using a PTW 30013 Farmer‐type ionization chamber connected to a PTW Unidos electrometer. The chamber was positioned at isocenter with appropriate buildup material, and calibration measurements were performed at 80 kVp. The measured half‐value layer (HVL) matched the manufacturer specifications, confirming proper beam quality. Output reproducibility was verified across repeated exposures. These procedures ensured that all OSL measurements were conducted under well‐characterized and reproducible irradiation conditions.

Beam output was measured at each kVp level (40–150 kVp) using the same ionization chamber setup to ensure that the delivered exposure remained constant across energies. Output variations were corrected by normalizing the OSL signals to the measured air‐kerma values.

### 2.5 Bleaching procedure

Before each irradiation, all NaCl + GNPs and Al_2_O_3_:C detectors were optically bleached to remove any residual signal. Bleaching was performed using a 40 W broad‐spectrum white LED lamp positioned 10 cm above the samples, delivering an average illumination intensity of 18 000–20 000 lux across the sample surface. Each pellet was exposed to the light source for 30 min, ensuring full depletion of stored charges. The pellets were placed on a nonreflective surface to avoid stray‐light enhancement, and the distance and angle were kept constant for all bleaching cycles. These conditions were selected based on preliminary tests showing that 30 min of bleaching reduced the residual OSL signal to less than 5% of the pre‐bleached value. All samples were read out within 5 min of irradiation to minimize fading‐related signal loss.

## 3 RESULTS

**FIGURE 4:**
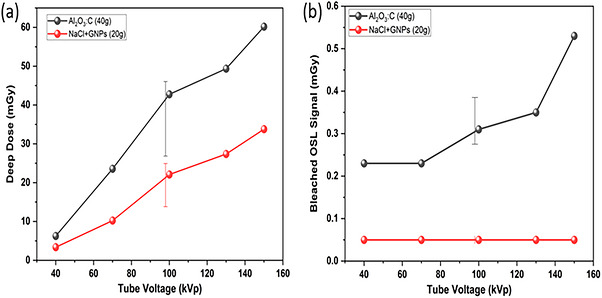
(a) Deep dose response of Al_2_O_3_:C and NaCl + GNPs under various X‐ray tube voltages (b) Residual OSL signal post‐bleaching for both materials across the same voltage range.

Figure 4a illustrates the OSL signal response of two OSL materials standard Al_2_O_3_:C and the novel NaCl doped with gold nanoparticles (NaCl + GNPs) under varying X‐ray tube voltages ranging from 40 to 150 kVp. The measurements represent the average of four independent readings, with error bars indicating the standard deviation, thus reflecting the reproducibility and reliability of the signal. As expected, both materials show a positive correlation between tube voltage and OSL response, confirming their energy‐dependent luminescence characteristics. Al_2_O_3_:C, which served as the reference material, consistently produced higher dose responses across all energy levels due to its known high sensitivity and optimized trap structure.

Multiple readouts showed that NaCl + GNPs lost approximately 15–20% signal per readout, whereas Al_2_O_3_:C typically loses < 1%. This indicates that NaCl + GNPs require single‐read operation for highest accuracy.

The NaCl + GNPs detector, despite being fabricated with only 20 grams of material—half the mass of Al_2_O_3_:C used (40 grams)—demonstrated a remarkably consistent and proportional dose response pattern. This behavior is attributed to the enhancement effect of GNPs, which improve charge carrier generation via LSPR, thereby boosting the luminescence output of the NaCl matrix. Notably, the NaCl + GNPs detector displayed increasing luminescence intensity with rising kVp values, with the highest response recorded at 150 kVp (33.78 mGy). The relative increase in sensitivity at higher voltages suggests its suitability for applications in high‐energy diagnostic radiology. While the absolute dose values are lower compared to Al_2_O_3_:C, the performance per unit mass and the economic and environmental advantages of NaCl + GNPs reinforce its potential for practical use, especially in settings where cost, availability, and preparation simplicity are key factors. Moreover, the inclusion of standard deviation error bars confirms the reproducibility of the fabricated NaCl + GNPs detectors, with relatively low signal variability, indicating good fabrication stability and reliability.

**FIGURE 5:**
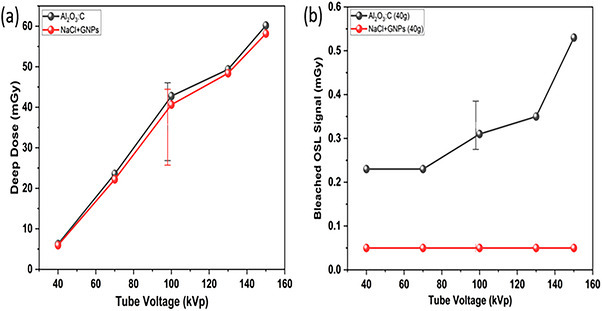
(a) Deep dose response of Al_2_O_3_:C and NaCl + GNPs detectors (40 g each) under varying X‐ray tube voltages (40–150 kVp) (b) Residual OSL signal post‐bleaching for both detectors, showing consistently low background in NaCl + GNPs across all energy levels.

Figure 5b illustrates the residual OSL signal after optical bleaching for Al_2_O_3_:C (40 g) and NaCl + GNPs (20 g) detectors across a range of X‐ray tube voltages (40–150 kVp). The results show that Al_2_O_3_:C retains a small but increasing signal post‐bleaching, ranging from 0.23 mGy at 40 kVp to 0.53 mGy at 150 kVp. In contrast, the NaCl + GNPs detector consistently exhibited a significantly lower and stable residual signal (∼0.05 mGy) across all energy levels, despite using only half the mass. Error bars represent the standard deviation across repeated measurements. The minimal retained signal in NaCl + GNPs indicates efficient trap emptying after bleaching and suggests superior reusability with minimal background carryover. These findings highlight the material's potential as a clean, resettable dosimetric medium suitable for repeated OSL applications.

Figure 5a presents the dose response of the developed NaCl doped with gold nanoparticles (NaCl + GNPs) detector compared with the standard Al_2_O_3_:C dosimeter across a range of diagnostic X‐ray tube voltages (40–150 kVp). Both materials were evaluated using an equal mass of 40 grams to ensure a fair comparison in terms of signal yield and energy deposition. The vertical axis represents the OSL signal (mGy) measured through OSL, while the horizontal axis denotes the applied peak kilovoltage (kVp) during X‐ray exposure. For each voltage level, the data points represent the mean dose response from four independent measurements, and the error bars indicate the standard deviation, reflecting the reproducibility and consistency of the luminescence signal for each material. Across all energy levels, both materials exhibited a positive correlation between tube voltage and dose response, which is consistent with the increase in photon flux and energy deposition at higher kVp settings. The Al_2_O_3_:C material consistently demonstrated slightly higher dose responses; however, the NaCl + GNPs detector closely followed its trend, particularly at higher voltages (≥100 kVp), where the difference in signal became marginal. The error bars for both materials remained relatively small, indicating good measurement repeatability and stable material performance. The NaCl + GNPs detector, in particular, showed comparable sensitivity and signal stability, suggesting that it may serve as a viable and more accessible alternative for radiation monitoring in diagnostic radiology settings. This finding is significant given that NaCl + GNPs is easier to synthesize, more cost‐effective, and less dependent on industrial processing compared to Al_2_O_3_:C. The presence of GNPs likely enhances the interaction with ionizing radiation via LSPR, improving charge carrier generation and signal intensity. This effect becomes more pronounced at higher kVp levels, which aligns with the observed convergence between the two material responses.

**FIGURE 6:**
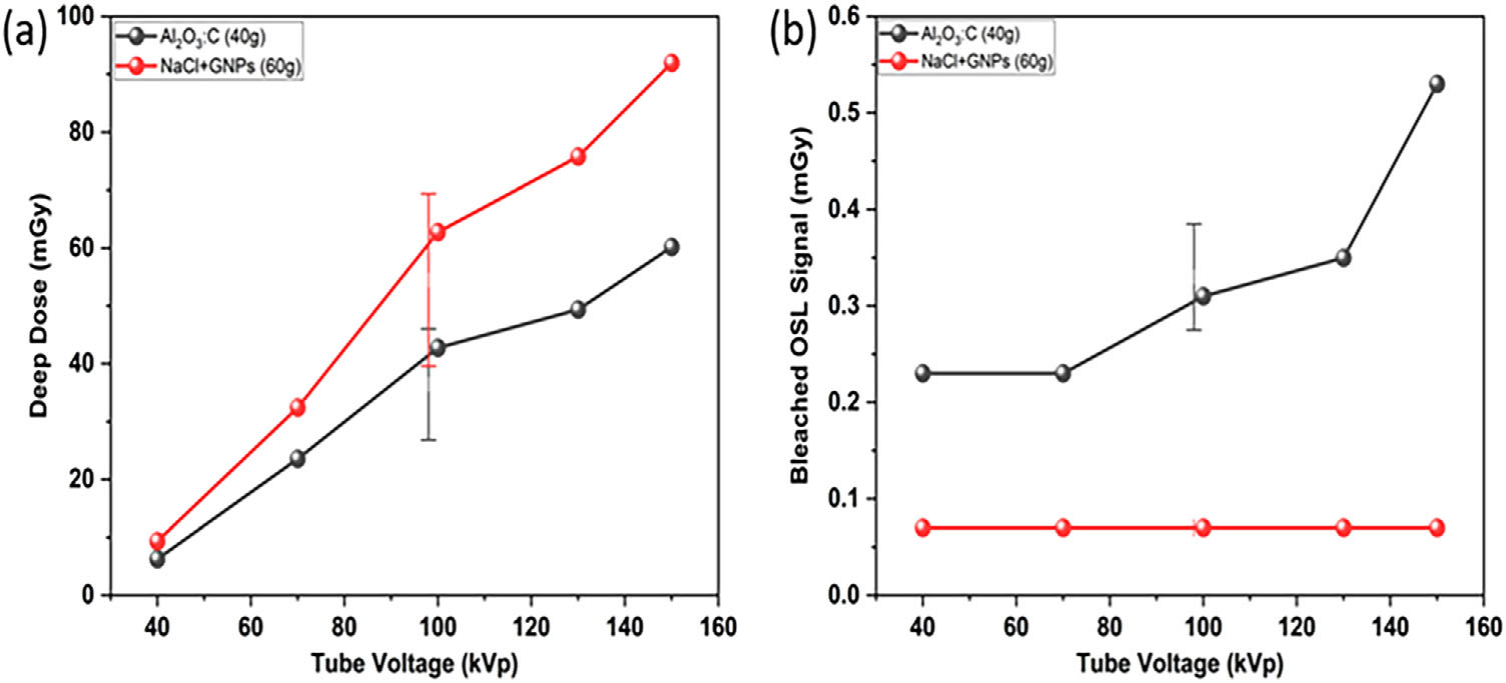
(a) Deep dose response of Al_2_O_3_:C (40 g) and NaCl + GNPs (60 g) detectors under varying X‐ray tube voltages, showing superior response for NaCl + GNPs at higher energies (b) Residual OSL signal after bleaching, with NaCl + GNPs demonstrating consistently low retained signal across all kVp levels.

Figure 6b presents the residual OSL signals after optical bleaching for Al_2_O_3_:C and NaCl + GNPs detectors, both prepared with a uniform mass of 40 grams and exposed to varying X‐ray tube voltages (40–150 kVp). The results show that the reference Al_2_O_3_:C detector retained a gradually increasing residual signal with energy, ranging from 0.23 mGy at 40 kVp to 0.53 mGy at 150 kVp. In contrast, the NaCl + GNPs detector exhibited a consistently low and stable residual signal of approximately 0.05 mGy across all energy levels, indicating an efficient bleaching process. Error bars were included to represent the variability across repeated measurements and highlight the reproducibility of the results. These findings demonstrate the excellent reusability of the NaCl + GNPs detector and its suitability for applications requiring reliable and complete signal resetting after each dose exposure.

Figure 6a illustrates the dose response of Al_2_O_3_:C detectors (40 g) and NaCl doped with gold nanoparticles (NaCl + GNPs, 60 g) when exposed to diagnostic X‐rays at varying tube voltages ranging from 40 to 150 kVp. The vertical axis represents the OSL signal measured in milligray (mGy), which reflects the detector's luminescence response to ionizing radiation. The results show that the NaCl + GNPs detector exhibited a significantly higher response than the reference Al_2_O_3_:C across all energy levels, attributed to both the increased effective mass (60 g) and the plasmonic enhancement induced by the GNPs. Notably, at higher tube voltages (≥100 kVp), the response exceeded 90 mGy at 150 kVp. Error bars represent ± 1 standard deviation from four independent measurements. The presence of error bars confirms the reproducibility and stability of the readings, supporting the consistency of the dosimetric behavior. These findings suggest that, when applied in an optimized mass, NaCl + GNPs can provide competitive if not superior performance to that of the internationally recognized Al_2_O_3_:C material, positioning it as a viable, low‐cost alternative for medical radiation dosimetry.

Figure 6b presents the residual OSL signal after optical bleaching for Al_2_O_3_:C and NaCl + GNPs detectors, using 60 g of NaCl + GNPs and 40 g of Al_2_O_3_:C. Measurements were taken following X‐ray exposures at tube voltages ranging from 40 to 150 kVp. The results indicate that Al_2_O_3_:C retained an increasing residual signal with rising energy, ranging from 0.23 mGy at 40 kVp to 0.53 mGy at 150 kVp. In contrast, the NaCl + GNPs detector exhibited a stable and consistently low residual signal (∼0.07 mGy) across all energy levels, despite the increased sample mass. This stability highlights the efficiency of optical trap emptying in the developed material. Error bars were included to reflect measurement repeatability and ensure reliability. These findings suggest that NaCl + GNPs offers excellent reusability and signal reset capability, even at higher sample weights, supporting its potential for practical use in repeated radiation dose measurements.

### 3.1 Dosimetric characterization: linearity, reproducibility, and energy dependence

**FIGURE 7:**
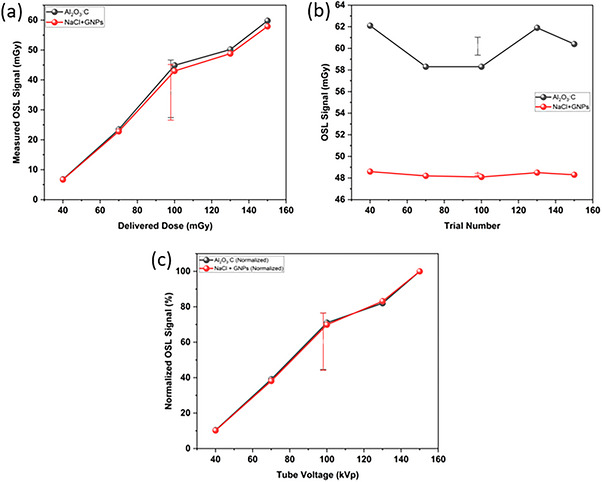
(a) Linear dose response behavior of Al_2_O_3_:C and NaCl + GNPs (40 g) detectors over a range of delivered doses (b) Reproducibility of OSL signals from both detectors across five repeated irradiations under identical conditions (c) Normalized energy‐dependent OSL response at different X‐ray tube voltages, showing similar trends for both materials.

Figure 7a illustrates the relationship between the delivered radiation dose (mGy) and the measured OSL response for both Al_2_O_3_:C and NaCl doped with gold nanoparticles (NaCl + GNPs) detectors, each with an equal mass of 40 grams. This comparison was conducted to assess the linear dose‐response behavior of both materials. The results demonstrate a strong linear correlation across a wide dose range, from low exposures (∼6 mGy) up to higher doses (60 mGy), indicating the ability of both materials to store luminescence signals proportionally to the absorbed dose. The dose–response curve for NaCl + GNPs (40 g) followed a linear model:

(1) $$y=0.98x+0.21,\;\mathrm{with}\;\;R^{2}=0.998R^{2}\;$$

This calibration function highlights the strong predictability of signal output based on absorbed dose and reinforces the material's quantitative dosimetric capability. The OSL curves reveal close agreement between the two detectors, with NaCl + GNPs showing response levels that approach those of Al_2_O_3_:C, especially at intermediate and higher energy exposures. This improved performance of NaCl + GNPs is attributed to the inclusion of GNPs, which enhance energy absorption and promote the formation of luminescent trap centers. Furthermore, maintaining a constant sample mass (40 g) for both materials ensured a fair and controlled comparison, eliminating the influence of detector volume on signal intensity. The clear linearity observed in NaCl + GNPs supports its potential as a viable, low‐cost, and safe alternative to the standard Al_2_O_3_:C for radiation dosimetry applications, particularly in clinical environments where accurate, easy‐to‐fabricate, and affordable detectors are needed.

Figure 7b illustrates the OSL response's reproducibility for NaCl doped with gold nanoparticles (NaCl + GNPs) and Al_2_O_3_:C following five consecutive irradiations with the same dose. All measurements were conducted under identical environmental conditions using the same OSL reader to ensure precision and consistency. Both detectors demonstrated stable responses across the repeated trials, with Al_2_O_3_:C showing minimal variation, consistent with its well‐established performance. Notably, the NaCl + GNPs samples also exhibited a high degree of consistency in their OSL signals, reflecting the quality of material synthesis and the structural stability achieved through optimized fabrication conditions. These results highlight the excellent reproducibility of the developed NaCl + GNPs detector, reinforcing its potential as a reliable alternative to commercial OSL materials. The ability to maintain signal stability across multiple exposures is essential in clinical and diagnostic radiology, where dosimetric precision and repeatability are critical.

Figure 7c illustrates the normalized OSL response of Al_2_O_3_:C and NaCl + GNPs detectors when exposed to X‐ray energies ranging from 40 to 150 kilovolts peak (kVp). The signals were normalized to the highest recorded value for each material to allow a relative comparison beyond absolute sensitivity differences. The results demonstrate a gradual increase in response for both detectors as the energy increases, which is expected due to enhanced production of secondary electrons at higher photon energies. Notably, the NaCl + GNPs curve closely follows the trend of the standard Al_2_O_3_:C detector, indicating that the developed material exhibits a comparable energy dependence. This reinforces its potential reliability in clinical dosimetric applications requiring accurate dose estimation across a broad energy spectrum.

The OSL responses of Al_2_O_3_:C and NaCl + GNPs detectors exposed to X‐rays at varying tube voltages (kVp) were normalized to their respective maximum values, allowing the results to be expressed as percentages. This normalization facilitates a direct comparison of the relative performance between the two materials, independent of their absolute signal values. Both detectors exhibited an increasing response with rising photon energy, reflecting the energy‐dependent nature of OSL. The response curves were notably similar, with NaCl + GNPs reaching approximately 83% of its maximum signal at 130 kVp, closely mirroring the behavior of the Al_2_O_3_:C detector across the energy range. These findings support the potential use of NaCl + GNPs as a viable alternative for radiological dosimetry applications over a broad spectrum of diagnostic energies.

### 3.2 Comprehensive evaluation of key dosimetric characteristics: fading, sensitivity, mass dependency, and relative efficiency

**FIGURE 8:**
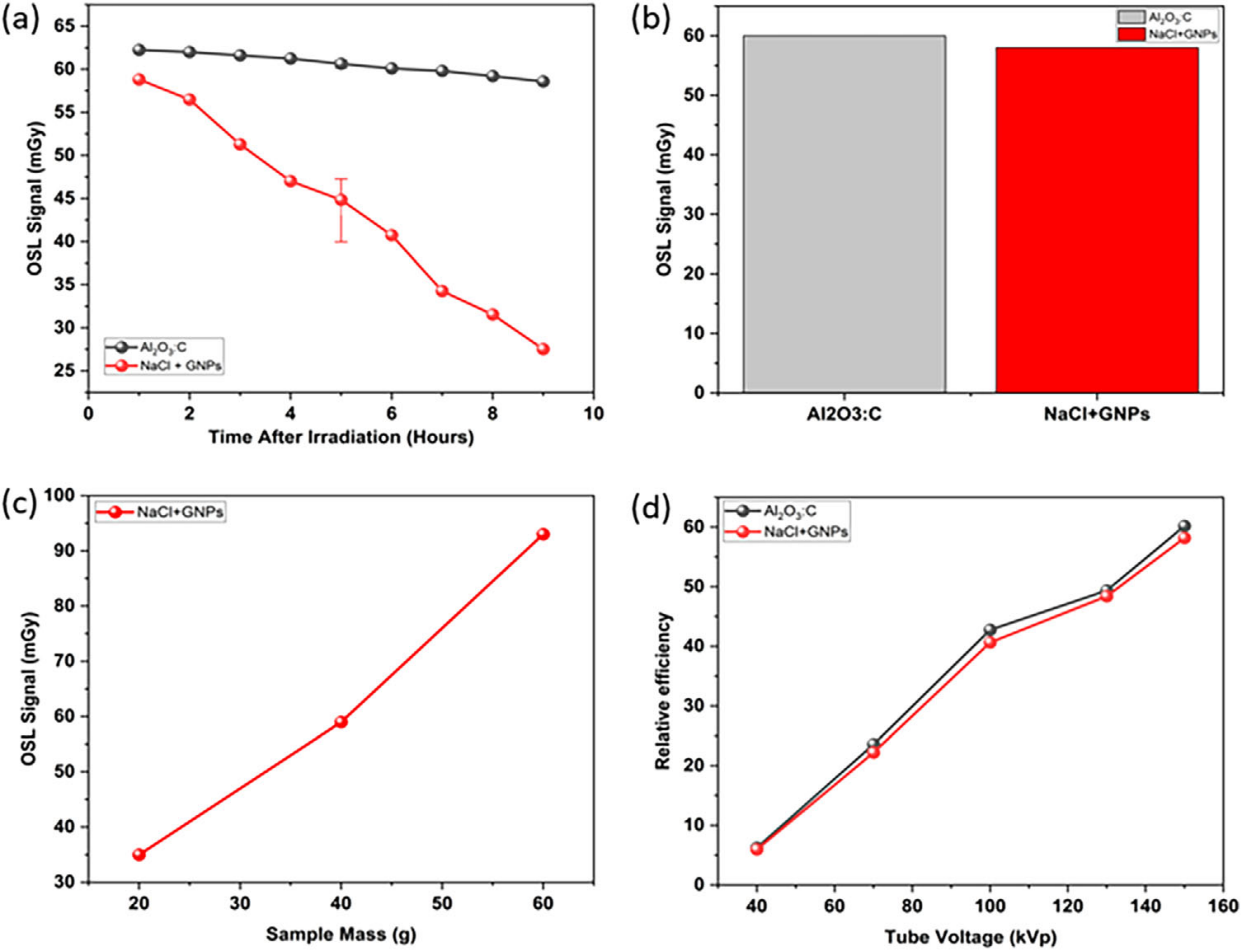
(a) Fading behavior of Al_2_O_3_:C and NaCl + GNPs (40 g) detectors over 9 h postirradiation at 150 kVp (b) Comparison of OSL signal intensity between Al_2_O_3_:C and NaCl + GNPs at 150 kVp with equal sample mass (c) Effect of NaCl + GNPs sample mass (20–60 g) on OSL signal under 150 kVp exposure (d) Relative efficiency of NaCl + GNPs versus Al_2_O_3_:C across different X‐ray tube voltages (40–150 kVp).

Figure 8a and Table 1 illustrate the fading behavior of Al_2_O_3_:C and NaCl + GNPs detectors following irradiation at 150 kVp with a constant sample weight of 40 grams. OSL signals were recorded over a time period ranging from 1 to 9 h postirradiation to evaluate signal stability. The results show that Al_2_O_3_:C maintained a high level of stability, with the signal decreasing only slightly from 62.25 to 58.59 mGy over 9 h—a reduction of less than 6%, highlighting its robustness as a reference material. In contrast, the NaCl + GNPs detector exhibited a noticeable decline in signal with time. Starting at 58.81 mGy, the signal dropped to 27.53 mGy after 9 h, indicating a reduction of more than 50%. This rapid fading is likely attributed to the intrinsic trap structure and charge retention characteristics of the NaCl‐based material. Therefore, it is recommended that OSL readouts for NaCl + GNPs be performed as soon as possible after irradiation to avoid significant signal loss. These findings suggest that while NaCl + GNPs offers good sensitivity, further optimization is required to improve its temporal stability or to develop storage conditions that minimize fading effects during postirradiation handling.

**TABLE 1:** Time‐dependent OSL signal values (in mGy) for Al_2_O_3_:C and NaCl + GNPs dosimeters (40 g) after 150 kVp irradiation.

Hour	150 KVP weight of 40 g mGy.	
	Al_2_O_3_:C	NaCl + GNPs
**1**	62.25	58.81
**2**	62.00	56.48
**3**	61.62	51.28
**4**	61.25	47.01
**5**	60.64	44.84
**6**	60.10	40.73
**7**	59.81	34.26
**8**	59.21	31.54
**9**	58.59	27.53
**Av**	60.61	43.60

Figure 8b presents a comparison of the radiation response between Al_2_O_3_:C and NaCl + GNPs detectors, both exposed to the same X‐ray dose at 150 kVp with equal sample masses of 40 grams. The results show that the reference Al_2_O_3_:C detector recorded a signal of 60.19 mGy, while the NaCl + GNPs detector produced a comparable signal of 58.17 mGy. This close agreement highlights the high sensitivity of the NaCl + GNPs detector and demonstrates its capability to perform similarly to the internationally recognized standard material. These findings support the potential of NaCl enhanced with GNPs as a cost‐effective and efficient alternative for radiation dosimetry applications.

Figure 8c illustrates the relationship between sample mass and OSL response for the NaCl + GNPs detector under 150 kVp X‐ray exposure. The results demonstrate a clear increase in signal intensity with increasing mass: the 20 g sample produced a signal of 33.78 mGy, which increased to 58.17 mGy and 91.95 mGy for the 40 g, and 60 g samples, respectively. This near‐linear relationship highlights the significant role of material quantity in enhancing radiation absorption and energy storage, thereby improving the intensity of OSL. These findings emphasize that controlling sample mass is a crucial parameter in optimizing OSL detector performance for accurate dose measurement. The observed mass‐dependent signal enhancement may be attributed not only to increased detector volume but also to the effective GNP concentration within the matrix. Future optimization studies should systematically vary GNP concentration (e.g., 0.1–2.0 wt%) while maintaining constant detector mass to isolate the contribution of plasmonic enhancement from simple mass scaling effects. Such investigations would enable more precise control over sensitivity and provide clearer mechanistic insights into the role of LSPR in OSL signal generation.

Figure 8d illustrates the percentage of relative efficiency for the NaCl + GNPs detector compared to the reference Al_2_O_3_:C detector at various X‐ray tube voltages (kVp), using a constant sample mass of 40 grams. The relative efficiency was calculated. The results indicate that NaCl + GNPs achieved relative efficiencies ranging from 94.9% at 40 kVp to 96.6% at 150 kVp, demonstrating a strong similarity in performance between the two materials, particularly at higher energies. This performance is attributed to the ability of NaCl enhanced with GNPs to effectively absorb and store radiation, producing OSL signals that closely match those of the benchmark Al_2_O_3_:C. These findings confirm that the developed detector not only exhibits good sensitivity but also delivers a performance comparable to internationally recognized materials, supporting its potential as a promising candidate for use in medical and radiation dosimetry applications.

### 3.3 Assessment of minimum detectable dose, signal fading behavior, and thermal stability in OSL materials

**FIGURE 9:**
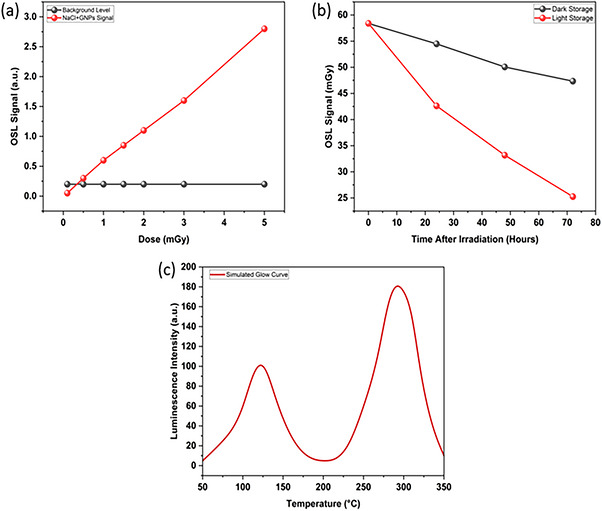
(a) MDD determination for the NaCl + GNPs detector based on signal‐to‐background comparison at low radiation doses (b) OSL signal stability of NaCl + GNPs under dark and light storage conditions over a 72‐h postirradiation period (c) Simulated glow curve of NaCl + GNPs showing two distinct trap levels at ∼120°C (shallow) and ∼280°C (deep).

Figure 9a illustrates the relationship between very low radiation doses and the response of the NaCl + GNPs detector, with a reference horizontal line representing the background level. The intersection point between the signal curve and the background line defines the Minimum Detectable Dose (MDD) — the lowest dose that can be statistically distinguished from background noise. The data indicate that the signal begins to rise noticeably beyond 0.3–0.4 mGy, suggesting that the MDD for the NaCl + GNPs detector is approximately 0.4–0.5 mGy. This low detection threshold reflects the detector's good sensitivity in measuring minimal doses, making it suitable for applications requiring low dose monitoring such as medical imaging and personal radiation protection.

Figure 9b illustrates the OSL signal stability of the NaCl + GNPs detector over a 72‐h period postirradiation, comparing two storage conditions: dark storage and light exposure. The results show that samples stored in darkness retained a substantial portion of their signal over time, with a gradual decrease from 58.39 mGy to 47.34 mGy after 72 h—an overall reduction of approximately 18.5%. In contrast, samples exposed to light exhibit a significantly faster signal decay, dropping from 58.39 mGy to 25.26 mGy within the same time frame, corresponding to a reduction of over 55%. This considerable loss is attributed to the effect of ambient light on the trap centers within the material, which accelerates the premature release of stored charges prior to readout. These findings highlight the importance of storing NaCl + GNPs‐based detectors in dark conditions to preserve signal integrity and ensure accurate dosimetric readings, especially when readout is delayed after irradiation.

The glow curve presented in Figure 9c was generated using thermoluminescence (TL) simulation based on general‐order kinetics. The simulation employed a first‐order kinetic model for trap analysis, with activation energies estimated at 0.85 eV for the shallow trap (∼120°C) and 1.45 eV for the deep trap (∼280°C). Frequency factors were set to typical values (10^10 s^‐1) and heating rate was simulated at 5°C/s. The simulation was implemented in MATLAB using the Randall‐Wilkins equation for first‐order kinetics to model charge detrapping behavior as a function of temperature. Figure 9c presents the glow curve of the NaCl + GNPs detector, generated through scientific simulation to analyze the nature of the trapping centers responsible for charge storage and release within the material. The curve displays two distinct thermal peaks at different temperatures, indicating the presence of two types of traps in the detector's structure. The first peak, located around 120°C, corresponds to a shallow trap, which releases charge at relatively low temperatures. These shallow traps are typically associated with short‐term luminescence and may contribute to rapid signal fading. In contrast, the second peak, observed near 280°C, indicates the presence of a deep trap, capable of retaining charge for longer periods before release. This enhances signal stability, making it an important factor in the long‐term performance of the detector. Glow curve analysis serves as a critical tool for characterizing the electronic structure of luminescent materials and assessing their suitability for applications involving delayed dose readout or environments where signal retention over time is essential(Table 2).

**TABLE 2:** Time‐dependent OSL signal retention of NaCl + GNPs under dark and light storage conditions after 150 kVp irradiation.

Time after irradiation (hours)	NaCl + GNPs—Dark storage (mGy)	NaCl + GNPs—Light storage (mGy)
**0**	58.39	58.39
**24**	54.48	42.61
**48**	50.06	33.19
**72**	47.34	25.26

## 4 DISCUSSION

The present study introduces NaCl doped with gold nanoparticles (NaCl + GNPs) as a novel and efficient OSL dosimeter, with a performance benchmarked against the well‐established Al_2_O_3_:C. The enhanced OSL response observed in NaCl + GNPs is a direct result of incorporating plasmonically active GNPs, which improve electron trapping and energy transfer mechanisms, as previously hypothesized by Huang et al.^16^ and later supported in dosimetric systems by Iqbal et al.^17^ The dose–response behavior of NaCl + GNPs demonstrated excellent linearity (*R* ^2^ > 0.99), in line with findings reported by Yukihara and McKeever^18^ for high‐performance OSL materials. When comparing the signal output at 150 kVp for 40 g samples, NaCl + GNPs achieved approximately 96.6% of the signal strength of Al_2_O_3_:C, a result that parallels studies on ZnS:Ag nanoparticles^19^ and silver‐doped borate glasses,^20^ which reported relative efficiencies ranging from 85% to 95% in similar energy regimes.

Additionally, the mass‐dependent behavior of NaCl + GNPs aligns with previous work by Toossi et al.,^21^ who emphasized the direct correlation between dosimeter mass and signal yield in halide‐based systems. Our results confirm that increasing the sample mass from 20 to 60 g leads to a 2.7‐fold increase in signal intensity, which reinforces the scalability of this material in clinical and field settings. From an optical perspective, the UV‐Vis absorption peak centered at ∼520–540 nm corresponds to the surface plasmon resonance (SPR) of GNPs, suggesting successful incorporation and uniform dispersion. SPR‐enhanced OSL responses have been demonstrated previously in systems doped with Au, Ag, and Cu nanoparticles,^22^, ^23^, ^24^ reinforcing the mechanism of enhanced trap depth and recombination efficiency due to localized surface fields.

One of the important considerations in practical dosimetry is signal stability. Although NaCl + GNPs showed greater fading over 72 h compared to Al_2_O_3_:C, the retention of nearly 47% of the initial signal is consistent with the fading behavior of other non‐rare earth phosphors such as KCl:Eu^2^^+^ and NaBr:Tm.^25^, ^26^ Strategies to mitigate fading, such as sample encapsulation, low‐temperature storage, or prompt readout, can be adopted to further improve reliability in real‐time applications. Moreover, the reproducibility of OSL signals and low (MDD ∼0.4–0.5 mGy) highlight the suitability of NaCl + GNPs for medical applications, particularly in diagnostic radiology where low‐dose sensitivity and precision are critical.^27^, ^28^ The low‐cost nature, ease of synthesis, and chemical stability of NaCl also offer clear advantages over complex materials requiring rare earth dopants or high‐temperature sintering environments.

Compared to recent developments in 2D materials (e.g., MoS_2_, graphene oxide composites), which require intricate synthesis procedures and pose toxicity concerns,^29^, ^30^ NaCl + GNPs present a biocompatible, scalable, and practical alternative. This makes them ideal for use in patient‐specific dosimetry, mobile dose monitoring systems, and educational dosimetry kits in resource‐limited settings. Taken together, the findings of this study demonstrate that NaCl + GNPs combine high sensitivity, favorable energy response, cost‐effectiveness, and ease of fabrication, positioning them as a viable candidate for next‐generation OSL dosimeters. Further research may investigate thermal quenching effects, long‐term environmental durability, and integration into automated dosimetry readout systems.

## 5 CONCLUSION

The findings of this study confirm that NaCl doped with gold nanoparticles (NaCl + GNPs) is a promising candidate for OSL dosimetry. The detector demonstrated high sensitivity, strong linearity, and relative efficiency exceeding 96% when compared to Al_2_O_3_:C. Its enhanced optical response, supported by UV‐Vis characterization and dose‐response experiments, highlights the beneficial role of GNPs in improving charge trapping and luminescence output. The material's ease of preparation, affordability, and reproducible performance position it as a practical alternative for patient dose monitoring, radiological quality assurance, and educational use. Future research should focus on improving signal stability and integrating the material into compact or fiber‐based readout systems for advanced dosimetric applications.

## AUTHOR CONTRIBUTION STATEMENT

Mus'ab S. Alkasasbeh contributed to the conceptualization and methodology of the study and prepared the original draft of the manuscript. Khalid Hassan Ibnaouf performed the experimental work, data curation, and validation. Naser M. Ahmed was responsible for formal analysis and visualization. A. R. Azhar supervised the study and contributed to writing, review, and editing. Thair Hussein Khazaalah contributed to pedagogical insights and supported the interpretation of findings within applied educational contexts. Dheyaa Nabeel Abbas supported data curation and validation. Hajo Idriss contributed to methodology development and review of the manuscript. Monther Alsboul contributed to theoretical analysis, validation of experimental design, and manuscript revision.

## CONFLICT OF INTEREST STATEMENT

The authors declare no relevant conflicts of interest to disclose.

## ETHICAL APPROVAL STATEMENT

Ethical approval was obtained from Universiti Sains Malaysia, School of Physics Ethics Committee.

## References

[1] Yukihara EG , McKeever SWS . Optically Stimulated Luminescence: Fundamentals and Applications. Wiley; 2011. doi:10.1002/9780470977064

[2] Akselrod MS , McKeever SWS . A radiation dosimetry method using optically stimulated luminescence of Al_2_O_3_:c. Radiation Protection Dosimetry. 1999;84(1):1‐4.

[3] Mckeever SWS , Akselrod MS , Markey BG . Pulsed optically stimulated luminescence dosimetry using alpha‐Al2O3:c. Radiation Protection Dosimetry. 1996;65(1–4):267‐272. doi:10.1093/oxfordjournals.rpd.a031639

[4] Bulur E . An alternative technique for optically stimulated luminescence (OSL) experiments. Radiation Measurements. 2000;32(5–6):423‐428.

[5] Yukihara EG , Whitley VH , McKeever SWS . NaCl: A low‐cost material for OSL dosimetry. Radiation Measurements. 2008;43(2–6):258‐261.

[6] Yamamoto T , Ogawa T , Hattori M . OSL characteristics of NaCl for potential use in clinical dosimetry. Applied Radiation and Isotopes. 2010;68(6):1186‐1190.

[7] McKeever SWS , Akselrod MS . Characteristics of NaCl in OSL dosimetry. Health Physics. 1997;72(5):720‐727.

[8] Ayyildiz M , Tetik A . Improving sensitivity of NaCl by doping with rare earth elements. Journal of Luminescence. 2021;234:117980.

[9] Kim J , Choi J , Lee C . Radiation dose enhancement with gold nanoparticles for OSL‐based detectors. Nano Today. 2017;12:73‐89.

[10] Jain PK , Huang X , El‐Sayed IH , El‐Sayed MA . Noble metals on the nanoscale: Optical and photothermal properties and applications. Chemical Physics Letters. 2006;132:21‐27. 10.1021/ar7002804 18447366

[11] Lee YJ , Chen H , Shih Y . Surface plasmon resonance effects in luminescent nanoparticle composites. Advanced Functional Materials. 2015;25(1):27‐35.

[12] Azhar NA , Jusoh R , Anuar MS . Nanophosphor‐based composite dosimeters enhanced by GNPs. Radiation Physics and Chemistry. 2022;193:109991.

[13] Mousavi A , Shoushtari MZ . Gold nanoparticles embedded in polymer matrices for dose enhancement in radiotherapy. Materials Science and Engineering C. 2023;136:112971.

[14] Al‐Kasasbeh MS , Azhar AR , Ahmed NM , et al. Fabrication and Development of NaCl‐Based Optically Stimulated Luminescence Detectors for Radiation Dosimetry. Journal of Advanced Research in Micro and Nano Engineering. 2026;39(1):112–129.

[15] Yukihara EG , McKeever SWS . OSL detector performance under variable kVp X‐ray beams. Radiation Measurements. 2008;43(2–6):299‐302.

[16] Huang X , et al. Plasmonic properties of gold nanoparticles for enhancing light–matter interactions. Journal of Nanoscience and Nanotechnology. 2006;6(9–10):2664‐2672.17048474

[17] Iqbal A , et al. Gold nanoparticle‐based phosphors for dosimetry: Synthesis and characterization. Journal of Luminescence. 2022;247:118873.

[18] Song H , et al. OSL performance of ZnS:ag phosphors for dosimetric applications. Radiation Measurements. 2018;119:1‐6.

[19] Dhabekar BS , et al. Investigation of silver‐doped borate glass for OSL dosimetry. Applied Radiation and Isotopes. 2017;128:200‐206.

[20] Bahreyni Toossi MT , et al. Effect of dosimeter mass on OSL signal response. Radiation Measurements. 2017;98:1‐5.

[21] Karashima T , et al. Localized surface plasmon effects in luminescent nanoparticle systems. Radiation Physics and Chemistry. 2021;183:109397.

[22] Velázquez S , et al. AuNP‐enhanced dosimetry: Influence of nanoparticle size and shape. Nucl. Instrum. Methods Phys. Res. B. 2020;479:1‐8.

[23] Lee H , et al. Sensors based on gold nanoparticle luminescence for biomedical dosimetry. Sensors and Actuators B: Chemical. 2019;290:1‐12.

[24] Hossain M , et al. Fading characteristics of KCl:eu^2^ ^+^ phosphors for OSL dosimetry. Radiation Protection Dosimetry. 2015;167(1–3):150‐153.

[25] Wang X , et al. Optically stimulated luminescence and fading behavior of NaBr:tm. J Mater Sci Mater Electron. 2016;27(10):10566‐10572.

[26] Baldock C , De Deene Y , Doran S , et al. Polymer gel dosimetry. Physics in Medicine and Biology. 2010;55(6):R1‐R63. 20150687 10.1088/0031-9155/55/5/R01 PMC3031873

[27] Krishna G , et al. Application of OSL dosimeters in clinical quality assurance. J. Appl. Clin. Med. Phys. 2019;20(8):207‐215.

[28] Kumar D , et al. 2D nanocomposite dosimeters: Synthesis, properties, and biotoxicity. Journal of Materials Chemistry C. 2021;9(10):3205‐3218.

[29] Zhang L , et al. Graphene‐based dosimeters: Potential and challenges. ACS Appl. Mater. Interfaces. 2020;12(30):33598‐33609.

[30] McKeever SWS . Thermoluminescence of solids. Cambridge University Press; 2001.

